# Supplementary material for: Osteology, relationships and functional morphology of Weigeltisaurus jaekeli (Diapsida, Weigeltisauridae) based on a complete skeleton from the Upper Permian Kupferschiefer of Germany
Source: PeerJ. 2021 May 20;9:e11413. doi: 10.7717/peerj.11413 (PMC8141288; doi:10.7717/peerj.11413)
Supplement: Supplemental Information 3 [file peerj-09-11413-s003.doc]

**Supplemental File S3 -**

The following changes were made to the character codings presented in Pritchard and Sues (2019):

*Petrolacosaurus kansensis* -

- 92. 0->1.

*Orovenator mayorum -*

- 13) ?->0.
- 22) ?->0.
- 23) ?->0.
- 36) ?->0.
- 37) ?->0.
- 41) ?->1.
- 57). ?->0.
- 58) 1->1/2
- 63) ?->0.
- 73) ?->0.
- 87) 1->?.
- 103) ?->0.
- 105) 1->0.
- 106) ?->0.
- 110) ?->0.
- 111) ?->
- 120) ?->0.
- 123) ?->0.
- 125) ?->0.
- 127) ?->0.
- 134) ?->0.

*Coelurosauravus elivensis -*

- 175) ?->0.
- 233) ?->1.
- 236) ?->1.
- 237) ?->0.
- 238) ?->0.
- 274) ?->0.
- 329) ?->0.

*Weigeltisaurus jaekeli -*

- 19) ?->0.
- 23) ?->0.
- 46) ?->1.
- 47) ?->0.
- 61) ?->0.
- 126) ?->0.
- 134) ?->0.
- 152) ?->0.
- 153) ?->1.
- 154) ?->1.
- 155) ?->0.
- 160) ?->1
- 175) ?->0.
- 178) ?->0.
- 179) ?->1.
- 188) ?->0.
- 203) ?->0.
- 233) ?->1.
- 234) ?->0.
- 235) ?->0.
- 292) ?->0.
- 317) ?->1.
- 326) ?->1.

*Megalancosaurus preonensis -*

- 51) 1->0.
- 304) 1->0.

*Vallesaurus cenensis* -

- 51) 1->0.
